# 大颗粒淋巴细胞白血病诊断与治疗中国专家共识（2026年版）

**DOI:** 10.3760/cma.j.cn121090-20260313-00130

**Published:** 2026-06

**Authors:** 中国抗癌协会血液肿瘤专业委员会 中华医学会血液学分会淋巴细胞疾病学组、红细胞疾病学组 中国惰性淋巴瘤协作组

## Abstract

大颗粒淋巴细胞白血病（LGLL）是主要发生在老年人群的、以细胞毒性淋巴细胞克隆性增殖为特点的疾病。近年来，LGLL的基础与临床研究，特别是新药治疗领域取得了巨大进展。为提高我国医务工作者对LGLL的诊断、鉴别诊断及规范化治疗水平，中国抗癌协会血液肿瘤专业委员会，中华医学会血液学分会淋巴细胞疾病学组、红细胞疾病学组和中国惰性淋巴瘤协作组制定了本版专家共识。

## Abstract (en)

Large granular lymphocytic leukemia（LGLL）is a disease primarily occurring in elderly populations, characterized by the clonal proliferation of cytotoxic lymphocytes. In recent years, significant progress has been made in both basic and clinical research on LGLL, particularly in the field of novel drug therapies. To improve the diagnostic, differential diagnostic, and standardized treatment levels of LGLL for medical professionals in China, Hematological Oncology Committee of China Anti-Cancer Association, Lymphoid Disease Group, Red Blood Cell Disease Group, Chinese Society of Hematology, Chinese Medical Association, Chinese Workshop of Indolent Lymphomas have jointly formulated this version of the expert consensus.

大颗粒淋巴细胞白血病（large granular lymphocytic leukemia，LGLL）是一种以无明显诱因出现外周血持续性克隆性大颗粒淋巴细胞增多（通常>0.5×10^9^/L）为特征的惰性淋巴细胞增殖性疾病，常侵犯外周血、骨髓和肝脾，淋巴结受累罕见。依据细胞来源分为T细胞LGLL（T-LGLL）和NK细胞LGLL（NK-LGLL）[1]。该疾病较为罕见，在亚洲人群中占慢性淋巴细胞增殖性疾病的6％，中国尚无大规模流行病学数据。由于LGLL相对少见且涉及疾病范围较广，临床诊断与治疗难度大，中国抗癌协会血液肿瘤专业委员会，中华医学会血液学分会淋巴细胞疾病学组、红细胞疾病学组以及中国惰性淋巴瘤协作组组织专家制订本共识，以规范我国医务工作者对本疾病的诊治。

一、共识制定方法

1. 共识目的：指导和促进各级各类接诊LGLL的临床医师规范地诊治LGLL。

2. 共识制定工作组：启动时间为2025年5月30日，定稿时间为2026年2月24日。本共识的制订工作组由血液科、病理科、流式细胞术检测实验室等多学科的专家组成。制订工作组成员按照主要职能分为首席专家、指导委员会、共识组和秘书组。所有制订工作组成员均填写了利益冲突声明表，均声明不存在与本共识相关的经济或非经济利益冲突。制订过程或推荐意见形成未受到资助影响。

3. 共识注册：本共识在制订初期撰写了计划书，并在国际实践指南注册与透明化平台（http://www.guidelines‑registry.cn）进行了中英文双语注册（注册号：PREPARE‑2025CN1212）。

4. 共识的使用者与目标人群：本共识的使用者为各级各类接诊LGLL的临床医师、药师、护理人员等相关医务工作者。目标人群为共识涉及的LGLL患者。

5. 证据检索与筛选：按照人群、干预、对照、结局（population，intervention，comparison，outcome，PICO）原则，制订包含主题词和自由词的检索策略并进行系统检索，检索范围包括：①PubMed、Embase、The Cochrane Library、Web of Science等英文数据库；②中国知网、维普、万方等中文数据库。检索时限涵盖建库至2025年5月30日，主要纳入系统评价、荟萃分析、随机对照试验、队列研究、病例‑对照研究、病例系列、病例报告和指南/共识等。完成文献检索后，共识专家组针对指南中的临床问题筛选文献，确定纳入符合具体临床问题的文献（发表语言限定为中、英文）。

6. 推荐意见的形成：指导委员会、秘书组及共识专家组基于国内外现有证据情况，同时考虑中国患者的偏好与价值观，干预措施的成本、利弊和可及性等，通过讨论形成符合我国临床诊疗实践的推荐意见。

7. 共识意见的撰写、外审及批准：推荐意见达成共识后，由执笔专家完成共识全文撰写，经所有共识专家组成员同意后，邀请外审小组进行同行评审，并根据外审意见进行修订与完善，最终提交指导委员会审议通过后确定共识终稿。

8. 共识发布与更新：本共识将通过专业期刊、网络及各类学术会议进行推广与发布，确保临床医师及其他利益相关群体充分了解本共识，促进临床医师和相关从业人员对共识内容的规范理解与合理应用。

二、临床表现及生物学特点

1. 临床特点：LGLL好发于中老年人，中位诊断年龄在60岁以上，仅10％患者为40岁以下，25岁以下患者罕见，中国LGLL患者的中位诊断年龄较西方国家更低，男女比例相当[2]。临床表现上，约1/3患者起病时无症状，2/3患者在疾病进展过程中出现贫血、脾大和中性粒细胞减少等临床症状，且常合并自身免疫性疾病、其他血液系统疾病或恶性肿瘤[3]。在西方国家，患者主要表现为中性粒细胞减少及反复感染。25％～30％的患者合并自身免疫性疾病，其中类风湿关节炎最常见，占LGLL的10％～18％[4]–[5]，还可合并其他自身免疫性疾病如系统性红斑狼疮和干燥综合征[6]–[7]。在我国，贫血症状更为突出，合并纯红细胞再生障碍（pure red cell aplasia，PRCA）的发生率约为20％[8]。超过30％的T-LGLL患者免疫学指标异常，如多克隆高γ球蛋白血症或抗核抗体阳性，提示免疫系统激活[9]。约1/4患者脾脏肿大，但肝脏及淋巴结肿大、淋巴瘤相关B症状（发热、盗汗、体重减轻）则较为少见。LGLL患者整体呈惰性表现，但其预期生存时间较健康人缩短，患者的中位总生存（OS）期为108.4个月[10]。

2. 免疫表型特点：T-LGLL通常表现为终末效应记忆T细胞表型（CD3^+^CD8^+^CD57^+^CD45RA^+^ CD62L^−^），NK-LGLL为CD3^−^CD16^+^或CD3^−^CD56^+^成熟NK细胞表型[11]。NK-LGLL中无症状患者较T-LGLL多，但患者接受免疫抑制剂治疗的疗效及OS无明显差异[12]–[13]。约30％的T-LGLL表达CD4，这类患者就诊时常表现为无症状性淋巴细胞增多，约50％的患者会出现STAT5B突变，并呈现更为惰性的病程[14]。西方国家的队列研究中，γδT-LGLL表现为更低的中性粒细胞、血红蛋白和血小板水平，更容易合并自身免疫性疾病及STAT3突变，OS期也更短[15]。然而，中国学者的研究数据表明，这两组患者的临床表现相似，OS期并无显著差异[16]。NK-LGLL内部仍具有异质性，CD56低表达或不表达患者更容易出现血细胞减少，需更积极治疗[17]。影响患者OS的主要因素是年龄，血小板减少、脾大、美国东部肿瘤协作组体能状态评分（ECOG评分）>2分、治疗线数>3也是患者预后不良的危险因素[2]。

3. 遗传学特点：STAT3突变是LGLL患者最常见的突变，突变率为11％～75％，是LGLL重要的致病基因突变。具有STAT3突变的LGLL患者无论为何种免疫表型，均呈现出明显的贫血、中性粒细胞减少，并与合并自身免疫性疾病、PRCA相关[18]。尽管STAT3突变患者疾病特点更具侵袭性，但往往对免疫抑制剂治疗更敏感[19]，且其OS与STAT3未突变患者无显著差异[13],[20]。表观遗传学调控相关基因突变是LGLL患者中第二常见的突变类型，包括TET2、ASXL1、DNMT3A等，其中TET2突变最常见（突变率为20％～30％）。合并TET2突变的NK-LGLL患者的PLT较未合并突变患者更低，且免疫抑制剂疗效更差[21]–[22]。

三、诊断与鉴别诊断

（一）WHO诊断标准

1. T-LGLL：符合3个必要标准，或2个必要标准+至少1个次要标准可诊断T-LGLL。

必要标准：

①符合典型的LGLL免疫表型：T-LGLL通常CD2^+^、CD3^+^、CD8^+^、CD57^+^、CD16^+^、CD56^−^，CD5和（或）CD7表达减低，多数表达TCRαβ，少部分表达TCRγδ[23]；

②克隆性检查阳性：流式细胞学TCRVβ或TRBC1阳性和（或）分子生物学TCR基因重排阳性；

③外周血大颗粒淋巴细胞（LGL）绝对计数升高（一般>0.5×10^9^/L）。

次要标准：

①骨髓免疫组化显示窦内细胞毒性T淋巴细胞浸润；

②检出特征性基因突变，如STAT3或STAT5B基因突变。

2. NK-LGLL：符合以下3个或以上主要标准，或至少2个主要标准加2个次要标准，可诊断NK-LGLL（见表1）[24]。

**表1:** NK细胞大颗粒淋巴细胞白血病（NK-LGLL）的诊断标准

NK-LGLL的诊断标准
主要标准
①NK细胞绝对计数≥1.0×10^9^/L；
②KIR限制性表达：以下两个或以上抗原表达减弱：CD158A<9％、CD158B<12％或CD158E<4％；
③CD94/NKG2A过表达；
④STAT3、STAT5B、CCL22或TET2突变而没有其他骨髓增生异常综合征相关改变。
次要标准
①其他免疫表型异常（如CD161低表达、活化KIR限制）；
②JAK-STAT、NF-κB、PI3K信号通路的其他基因突变；
③骨髓活检显示NK细胞窦间隙浸润；
④无骨髓增生异常改变或骨髓增生异常综合征相关性分子异常^a^。

**注** ^a^骨髓增生异常综合征相关性分子异常：ASXL1、BCOR、EZH2、RUNX1、SF3B1、SRSF2、STAG2、U2AF1和（或）ZRSR2突变，复杂核型和（或）del（5q）、−7/del（7q）、+8、del（12p）、del（17p）、del（20q）或idic（X）（q13）

3. 诊断标准补充说明：

①既往的诊断标准要求外周血LGL绝对计数>2.0×10^9^/L，但有较多患者由于LGL浸润骨髓或伴随自身免疫性疾病出现血细胞减少症，此部分患者的LGL克隆扩增较低，所以诊断标准更新LGL绝对计数阈值为0.5×10^9^/L。如果患者外周血LGL绝对计数<0.5×10^9^/L且无症状，6个月后需复查LGL表型及克隆性。肿瘤细胞绝对值<1.0×10^9^/L的患者，建议完善骨髓检查以除外合并其他血液系统疾病可能[4]。

②LGL体积较大（直径15～18 µm），富含胞质，其中有典型嗜天青颗粒，偶见无颗粒型，细胞核呈肾形或圆形。形态不是诊断LGLL的必要条件，但患者表现为高白细胞、肝脾肿大、侵袭型病程时，需要结合形态进一步与T细胞幼淋巴细胞白血病进行鉴别。

③对于NK-LGLL，在没有确切克隆性标志时，NK细胞增多持续>6个月，同时除外可能的刺激因素对其诊断很重要。

④对于流式细胞术无法确定克隆性的NK细胞增多，可参考克隆性评分（NK细胞绝对计数>1.0×10^9^/L，2分；KIR限制性表型，2分；CD94或NKG2A高表达，1分；STAT3、STAT5B、TET2或TNFAIP3突变，2分），评分≥4分诊断NK-LGLL的敏感性达83％，特异性达96％[25]。

（二）鉴别诊断

1. 反应性LGL增多：多种疾病可出现LGL增多，如脾切除术、异基因造血干细胞移植术后、实体器官移植术后、感染（如人类免疫缺陷病毒、EB病毒、巨细胞病毒、乙型肝炎病毒）、自身免疫性疾病、血液病［如淋巴瘤、骨髓增生异常综合征（MDS）］及其他实体肿瘤等。LGL克隆性检查为LGLL和反应性LGL增多的主要鉴别点，LGL多数为多克隆性或寡克隆性，且仅持续数月，不导致血细胞减少。在诊断困难的情况下，骨髓活检有助于鉴别，反应性LGL增多常不伴LGL骨髓浸润。

2. 意义未明的T细胞克隆（T-CUS）：T-CUS是一种持续存在的克隆性T细胞扩增状态，在分子学和免疫表型特征方面与T-LGLL高度相似，但缺乏支持T细胞肿瘤诊断的临床（如贫血、粒细胞减少等）或实验室特征，可能是一种前驱状态。LGL的KLRG1分子表达缺失可能有助于鉴别（T-LGLL患者的LGL多为KLRG1表达阴性）[26]。

3. 再生障碍性贫血（AA）：约5％的LGLL患者有全血细胞减少，伴骨髓衰竭表现，甚至符合AA的临床标准。此时，可能难以区分特发性AA和伴全血细胞减少的LGLL。但外周血或骨髓中如持续存在大量克隆性增殖的LGL，则更支持LGLL的诊断。

4. 侵袭性NK细胞白血病：是一种EB病毒相关的NK细胞恶性增殖所致全身性疾病，好发于30～50岁中年人，多见于亚洲人。患者起病急，常伴有多部位、多器官受累，发热、系统性症状常见，病程呈侵袭性。典型免疫表型为：CD2^+^、膜表面CD3^−^、胞质CD3ε^+^、CD16^+^、CD56^+^，而CD57^−^。

5. 其他T细胞淋巴瘤白血病期：T细胞幼淋巴细胞白血病、成人T细胞淋巴瘤/白血病、塞扎里综合征（SS）等成熟T细胞淋巴瘤侵犯外周血时需要与LGLL进行鉴别，其细胞形态、免疫表型、病毒感染和组织浸润特点等均与LGLL存在差异。

（三）检查

治疗前（包括复发患者）应对患者进行全面评估，应至少包括：

1. 病史：包括详细的既往史、合并症，特别是自身免疫性疾病病史；体格检查，主要是肝脾肿大和淋巴结肿大。

2. 体能状态评分：如ECOG评分。

3. B症状：盗汗、发热、体重减轻。

4. 血常规检查：包括WBC及分类、PLT、HGB、网织红细胞百分比、网织红细胞绝对值、外周血涂片等。

5. 血生化检查：肝肾功能、乳酸脱氢酶（LDH）、β_2_微球蛋白等。

6. 免疫学检查：①免疫球蛋白定量：至少包括IgM、IgA、IgG水平；②自身抗体检查：抗核抗体、抗可提取性核抗原（ENA）抗体谱、抗磷脂抗体、风湿三项；③红细胞沉降率等。

7. 流式细胞术检查：LGL免疫表型检测（包括CD3、CD4、CD8、CD16、CD45RA、CD45RO、CD56、CD57、TCRαβ、TCRγδ、Perforin、Granzyme B等）、TCR Vβ和KIR检测；淋巴细胞亚群（B/T/NK细胞）。

8. 分子生物学检查：外周血TCR基因重排，二代测序检测，至少包括STAT3、STAT5B、TNFAIP3、CCL22、TET2基因等。

9. 病理检查（必要时）：骨髓活检+涂片+免疫组化+流式细胞术分析。

10. 影像学检查（选做）：肝脾B超，颈、胸、全腹部增强CT检查。

11. 其他可做的检查包括：直接抗人球蛋白试验、结合珠蛋白检测、游离血红蛋白检测（怀疑有溶血时必做）等。对于NK-LGLL，应做血EB病毒检测。

四、治疗

（一）治疗指征

对于无症状LGLL患者，采取随诊观察策略，每3～6个月随访1次。治疗指征包括[27]：①中性粒细胞计数<0.5×10^9^/L，或中性粒细胞计数<1.5×10^9^/L伴反复感染；②HGB<100 g/L，出现症状性贫血表现，或需要输注红细胞维持；③PLT<50×10^9^/L；④合并需要治疗的自身免疫性疾病；⑤症状性脾大；⑥明显的B症状；⑦肺动脉高压。

（二）治疗选择

LGLL的一线治疗方案为免疫抑制剂，包括环磷酰胺（CTX）、甲氨蝶呤（MTX）及环孢素A（CsA）；糖皮质激素可以作为中度全血细胞减少患者的补充治疗短期联合使用，血象改善后应迅速减停；也推荐患者尝试参加设计良好的临床试验。我国学者发现，以免疫调节剂沙利度胺为基础的TPM方案（沙利度胺+MTX+泼尼松）显示出良好的疗效，可以考虑作为LGLL的一线治疗。一线治疗失败或复发后，可选择交叉使用另一种免疫抑制剂；抗淋巴细胞球蛋白、JAK2抑制剂芦可替尼、抗CD52单抗、氟达拉滨等作为二线治疗也被证实具有一定疗效。新药临床试验也可作为二线选择，如果仍无效，可考虑造血干细胞移植。目前LGLL治疗的用药时长无统一意见，但普遍认为CTX的应用时间不宜超过1年，获得缓解的患者中约3/4可获得无药物持续缓解[28]。治疗推荐流程见图1。

**图1:**
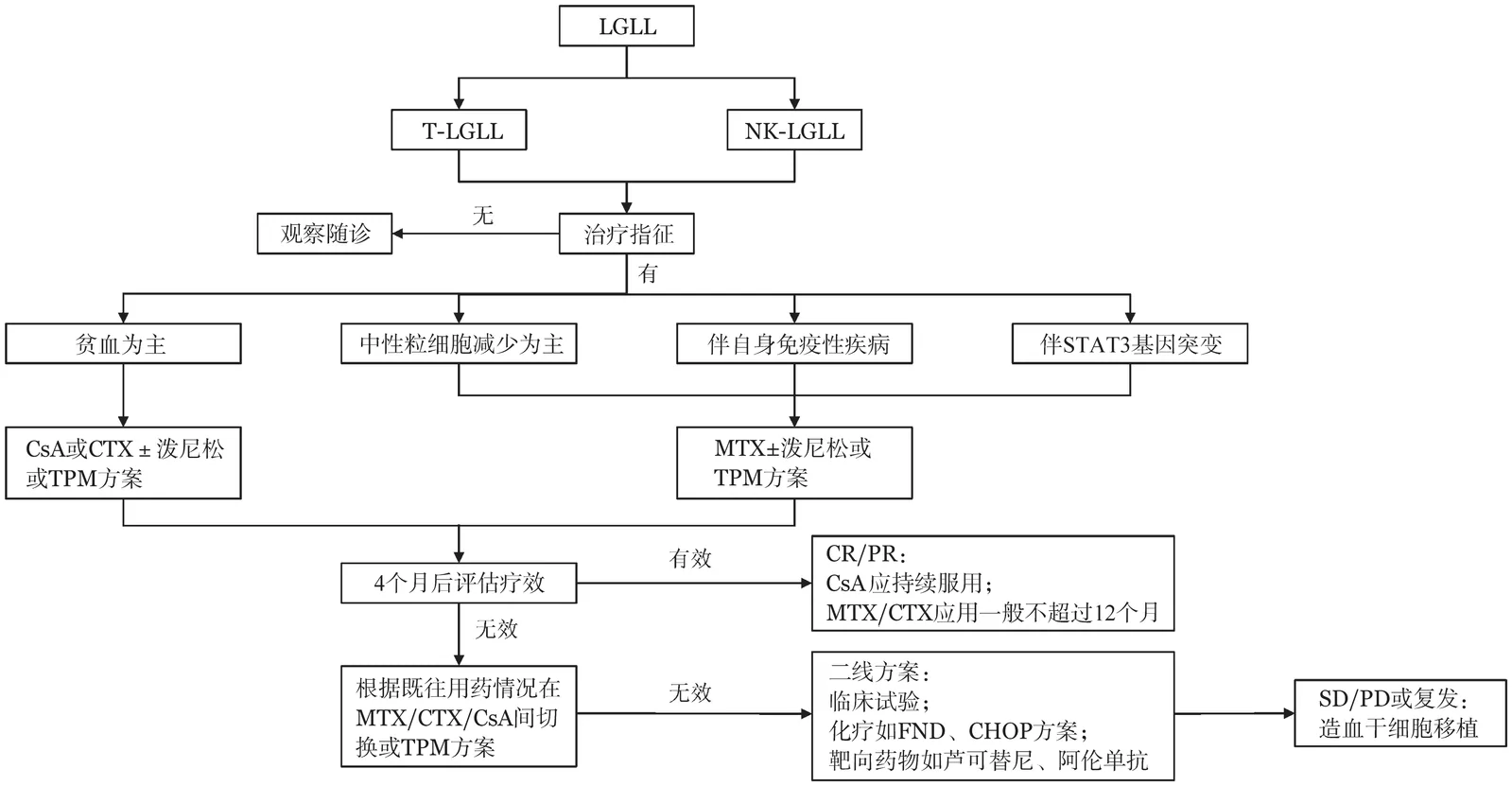
大颗粒淋巴细胞白血病（LGLL）治疗推荐流程 **注** T-LGLL：T细胞LGLL；NK-LGLL：NK细胞LGLL；CsA：环孢素A；CTX：环磷酰胺；TPM方案：沙利度胺+甲氨蝶呤+泼尼松；MTX：甲氨蝶呤；CR：完全缓解；PR：部分缓解；FND方案：氟达拉滨+米托蒽醌+地塞米松；CHOP方案：环磷酰胺+阿霉素+长春新碱+泼尼松；SD：疾病稳定；PD：疾病进展

（三）治疗方案

目前针对LGLL的治疗选择包括以下几个方面：免疫抑制治疗、免疫调节剂治疗、细胞毒性药物化疗、靶向治疗和造血干细胞移植。

1. 免疫抑制剂治疗：免疫抑制剂是目前治疗LGLL的主要药物，主要包括MTX、CTX、CsA，各方案的疗效见表2。

**表2:** 大颗粒淋巴细胞白血病一线治疗方案的疗效

方案	作者	病例数	客观缓解率	完全缓解率
甲氨蝶呤	Lamy等[29]	76	24％	12％
	Dong等[6]	89	65％	16％
	Sanikommu等[30]	61	43％	–
	Qiu等[31]	36	86％	22％
	Loughran等[19]	55	38％	5％
	Bareau等[32]	62	55％	21％
环磷酰胺	张乐乐等[28]	36	69％	53％
	Lamy等[29]	74	68％	46％
	Dong等[6]	65	62％	32％
	Sanikommu等[30]	53	53％	–
	Loughran等[19]	14	64％	21％
	Bareau等[32]	32	66％	47％
环孢素A	Dong等[6]	39	74％	23％
	Zhu等[8]	99	49％	20％
	Sanikommu等[30]	74	49％	–
	Bareau等[32]	24	21％	4％
沙利度胺+泼尼松+甲氨蝶呤	Yu等[33]	52	90％	75％

**注** –：无数据

（1）MTX：通常用法为10 mg/m^2^，每周1次，有效率为24％～65％，完全缓解（CR）率为5％～22％，中位起效时间为1～4个月[6],[19],[29]–[32]，故MTX的疗效评估一般在用药4个月后进行。以中性粒细胞减少为主要表现的患者更容易从MTX治疗中获益[19]。伴STAT3突变患者接受MTX的血液学缓解率高于不伴STAT3突变者，且疗效持续时间更长[19],[30]。MTX的药物不良反应包括肝肾功能异常、胃肠道反应、肺毒性、中枢神经系统毒性等，应用MTX的同时可每日补充5 mg叶酸。

（2）CTX：通常用法100 mg/d，主要用于以贫血为主要表现的患者，客观缓解率（ORR）为52％～68％，CR率最高为47％[6],[19],[29],[32]。2024年美国血液学年会上公布了CTX与MTX的Ⅱ期随机对照临床试验结果，显示CTX的疗效优于MTX（ORR：69％对24％，CR率：46％对12％）[29]。我国一项纳入37例T-LGLL患者的单中心回顾性研究显示，CTX血液学缓解率为69.4％，中位达血液学缓解的时间为2（0.75～7）个月[28]。目前推荐CTX应用4个月后进行疗效评估，无效者停用；有效者最长使用不超过12个月，以降低膀胱毒性、继发第二肿瘤的风险。CTX停药后患者依然可以保持长时间无治疗缓解，上述回顾性数据显示，中位随访39（8～56）个月，CTX停药后中位无治疗缓解（TFS）时间未达到，预估12个月TFS率为90.87％，36个月TFS率为75.72％。

（3）CsA：通常为3～5 mg·kg^−1^·d^−1^；或固定剂量50～100 mg/次，每日2次，根据患者治疗反应调整用量。CsA的有效率为21％～74％，CR率为4％～23％，与MTX的反应率相当，贫血尤其是继发PRCA患者疗效更佳[6],[8],[30],[32]。LGLL接受CsA治疗停药后容易复发，常需长期服用，用药期间需要监测其不良反应，如肾功能损害、血压升高等。CsA对于表达HLA-DR4患者的有效率可能更高[34]。

（4）其他免疫抑制治疗：对于复发难治患者，中国医学科学院血液病医院曾尝试应用抗胸腺细胞球蛋白（3～4  mg/kg，每天1次，持续应用5 d）治疗10例复发/难治LGLL，其中50％患者有效，40％患者达CR[8]。

2. 免疫调节剂治疗：LGLL的疾病特征之一是免疫系统失调，且经常与自身免疫性疾病同时发生[35]。中国医学科学院血液病医院淋巴瘤诊疗中心设计了一项以免疫调节剂为基础的联合用药方案：TPM方案，具体用法为沙利度胺100 mg每晚1次，推荐最长持续使用2年；MTX每周10 mg/m^2^，推荐使用1年；泼尼松0.5～1.0 mg/kg，隔日1次，3个月后逐渐减停，最长使用1年。共52例LGLL患者可评估疗效，有效率达90.4％，CR率达75.0％，高于目前报道的以免疫抑制剂为主的治疗方案[33]。该方案对贫血改善明显，83％患者治疗后HGB可恢复正常，在合并PRCA和伴STAT3突变患者中均有较好疗效，可作为LGLL的一线首选治疗。该方案可能出现的不良反应为周围神经病变、深静脉血栓形成等，治疗期间需加强监测。

3. 细胞毒性药物联合化疗：细胞毒性药物联合化疗一般仅限于免疫抑制治疗无效的患者，如CHOP样方案。核苷类似物如喷司他丁、克拉屈滨和氟达拉滨有应用于LGLL的报道，多数为个案报道，总有效率为79％[3]。我国香港的一项研究报道，氟达拉滨联合米托蒽醌和地塞米松（FND方案）治疗伴PRCA的T-LGLL，总有效率为100％，CR率为67％[36]。大剂量CTX（30 mg/kg，每日1次，持续应用4 d）也是有效的治疗方案之一，小样本研究显示，大剂量CTX治疗复发难治LGLL的ORR为78％，CR率可达56％[8]。

4. 新药治疗：依据T-LGLL发病机制，推测可能有效的新药包括单克隆抗体如抗CD52单抗，小分子靶向药物如Ras抑制剂、JAK抑制剂、PI3K抑制剂、蛋白酶体抑制剂、细胞因子拮抗剂和针对表观遗传修饰的药物等[37]–[38]。但目前接受此类药物治疗的LGLL例数较少，需更大规模前瞻性临床试验进一步验证其疗效和远期预后。新药治疗方案的有效率见表3。

**表3:** 大颗粒淋巴细胞白血病新药治疗方案的疗效

类型	药物	机制	病例数	客观缓解率	完全缓解率
JAK/STAT通路	芦可替尼（Ruxolitinib）[39]	JAK1/2抑制剂	21	86％	14％
	托法替尼（Tofacitinib）[40]	JAK3抑制剂	9	89％	0
PI3K/Akt通路	林普利塞（Linperlisib）[26]	PI3Kδ抑制剂	8	88％	50％
细胞毒性T细胞单抗	阿伦单抗（Alemtuzumab）[41]	CD52单抗	25	56％	36％
	阿巴西普（Abatacept）[30]	CTLA4单抗	8	75％	–
B细胞单抗	利妥昔单抗（Rituximab）[42]	CD20单抗	14	100％	79％
细胞因子拮抗剂	BNZ-1[43]	IL-15/2/9拮抗剂	14	20％	0
	人源化Mik-Beta-1单抗[44]	IL-2/IL-15β（CD122）拮抗剂	9	0	0
表观遗传修饰	贝利司他（Belinostat）[45]	HDAC抑制剂	1	100％	0
	地西他滨（Decitabine）[46]	DNMT抑制剂	1	100％	0

**注** –：无数据

（1）戈利昔替尼（Golidocitinib）：JAK/STAT通路失调是LGLL的致病标志。约60％的T-LGLL和30％的NK-LGLL患者出现STAT3功能获得性突变。戈利昔替尼是一种高选择性JAK1抑制剂，2025年美国血液学年会报道，戈利昔替尼治疗复发难治LGLL的ORR达92％（12/13），CR率为62％（8/13）[47]。

（2）林普利塞（Linperlisib）：是一种高选择性PI3Kδ抑制剂，目前主要用于复发难治的滤泡性淋巴瘤、慢性淋巴细胞白血病和T细胞淋巴瘤的治疗[48]。小样本研究尝试应用林普利塞治疗T-LGLL合并PRCA患者，4例患者均治疗有效，其中3例患者达到CR[49]。在一项Ⅰ期研究中，8例复发难治LGLL患者接受林普利塞治疗，有效率达87.5％[26]。林普利塞起效较快，中位起效时间仅2周，应用后红细胞生成可显著改善。

（3）芦可替尼（Ruxolitinib）：是一种JAK1/2抑制剂，可通过抑制JAK/STAT信号通路发挥抗肿瘤作用。一项多中心回顾性临床研究纳入21例复发难治LGLL患者，ORR为85.7％，CR率为14.3％[39]。

（4）利妥昔单抗（Rituximab）：作为抗CD20单抗，利妥昔单抗广泛应用于B细胞淋巴瘤，并有显著的疗效。一项回顾性研究显示，14例合并类风湿关节炎的LGLL患者接受利妥昔单抗治疗的ORR为100％，CR率为79％，CR患者不仅血常规恢复正常，LGL绝对计数也≤0.3×10^9^/L。药物起效机制可能与利妥昔单抗减低IL-10水平、抑制B细胞的抗原提呈作用及杀伤CD20^+^T细胞相关[42]。

（5）托法替尼（Tofacitinib）：是一种作用于JAK的新型口服小分子抑制剂，主要抑制JAK1和JAK3，轻度抑制JAK2。LGLL常伴JAK-STAT信号通路异常，故靶向JAK/STAT通路信号分子可能有效。托法替尼已被美国食品药品监督管理局批准用于复发难治类风湿关节炎和溃疡性结肠炎，故伴类风湿关节炎或其他自身免疫性疾病的LGLL可能更适合接受该药治疗[50]。小样本的临床数据显示，接受托法替尼单药治疗的复发难治LGLL患者的ORR可达89％[40]。

（6）阿伦单抗（Alemtuzumab）：是一种人源化抗CD52单克隆抗体，一项早期纳入25例患者的小样本临床试验结果报道，其总体有效率为56％，4例合并MDS和2例移植后患者均无治疗反应，除外上述6例患者，经典LGLL接受阿伦单抗的ORR为74％。但阿伦单抗的输注相关不良反应值得注意，此外，该药会造成骨髓抑制，导致感染风险增加，包括巨细胞病毒感染和再活化[41]。

（7）细胞因子拮抗剂：研究表明，IL-15可通过促进肿瘤细胞抑癌基因甲基化、激活多条生存信号通路（如JAK/STAT、Ras、PI3、NK-κB）促进LGL增殖[51]。细胞因子拮抗剂BNZ-1是一种聚乙二醇化的19聚体γc结合肽，可以通过阻断细胞因子与其受体的结合选择性抑制IL-2、IL-9和IL-15。一项Ⅰ/Ⅱ期临床试验将其应用于初治或复发难治LGLL患者，ORR仅为20％。

5. 造血干细胞移植：造血干细胞移植不是T-LGLL的常规治疗方式，仅在患者对多数药物耐药情况下考虑应用。一项小样本的回顾性研究报道了接受造血干细胞移植治疗的LGLL患者的疗效，10例患者接受异基因造血干细胞移植（allo-HSCT），5例接受自体造血干细胞移植（auto-HSCT）。allo-HSCT患者的CR率可达70％，部分患者可获得持续缓解，但移植后死亡率较高，40％患者因移植并发感染死亡。接受auto-HSCT患者的CR率达30％[52]。

6. 脾切除术：脾大伴疼痛或伴脾梗死出血患者可考虑脾切除术。早期有研究以脾切除术作为LGLL的治疗手段，约56％患者可出现治疗反应[3]，但均为血细胞异常短暂缓解，故不推荐作为LGLL的常规治疗手段。

（四）辅助治疗

1. 粒细胞集落刺激因子（G-CSF）：LGLL治疗前单纯应用G-CSF有效率不高，或仅短暂有效，粒细胞减少伴发热患者可考虑应用，但可能会加重脾大或关节疼痛，需要注意。

2. 促红细胞生成素（EPO）：有报道显示部分患者对EPO短暂有效，CsA治疗有效后联合EPO可能使部分患者疗效进一步提升。但对于继发PRCA患者不建议应用。

五、疗效评价

由于LGLL的治疗起效缓慢，建议至少充分治疗4个月后评估疗效，无效者再考虑更换方案，疗效标准参考表4[53]–[54]。

**表4:** 大颗粒淋巴细胞白血病（LGLL）的疗效评价标准

治疗反应	评价标准
分子学完全缓解（CMR）	达CR同时检测不到克隆性LGL（应用流式细胞术或PCR检测TCR重排阴性）
血液学完全缓解（CR）	临床症状体征正常（脾肿大消失）且血细胞计数完全正常：即HGB≥110 g/L（女性）或120 g/L（男性），PLT>100×10^9^/L，ANC>1.5×10^9^/L，淋巴细胞计数<4.0×10^9^/L，外周血LGL在正常范围内（<0.5×10^9^/L）
血液学部分缓解（PR）	HGB>80 g/L或较基线增加≥20 g/L并脱离红细胞输注；ANC>0.5×10^9^/L，无反复感染；PLT>50×10^9^/L并脱离血小板输注
疾病稳定（SD）	规范治疗4个月后达不到CR/PR标准，但未出现明显进展
疾病进展（PD）	血细胞减少或器官肿大进行性加重

**注** LGL：大颗粒淋巴细胞；PCR：聚合酶链反应；TCR：T细胞受体；HGB：血红蛋白；PLT：血小板计数；ANC：中性粒细胞绝对计数

六、随访

无症状患者建议每3～6个月随访1次，疗效达部分缓解及以上患者结束治疗后，第1年建议每3个月随访1次，以后每3～6个月随访1次。随访的主要内容包括：病史（特别是自身免疫性疾病病史和肿瘤病史）、查体（特别是肝脾肿大和淋巴结肿大情况）、实验室检查（主要是血常规+网织红细胞百分比及绝对值、LGL免疫分型）。
